# Characterisation and sequencing of the novel phage Abp95, which is effective against multi-genotypes of carbapenem-resistant *Acinetobacter baumannii*

**DOI:** 10.1038/s41598-022-26696-9

**Published:** 2023-01-05

**Authors:** Li Huang, Siyi Huang, Lingli Jiang, Jingjie Tan, Xueping Yan, Chunmei Gou, Xinchong Chen, Lijuan Xiang, Dali Wang, Guangtao Huang, Yixin Zhang, Chengliang Deng

**Affiliations:** 1 Department of Burn and Plastic Surgery, The Affiliated Hospital of Zunyi Medical University, Zunyi, 563000 China; 2 Department of Laboratory Medicine, The First Affiliated Hospital of Shenzhen University, Shenzhen Second People′s Hospital, Shenzhen, China; 3 Department of Burn and Plastic Surgery, Department of Wound Repair, Shenzhen Institute of Translational Medicine, The First Affiliated Hospital of Shenzhen University, Shenzhen Second People’s Hospital, Shenzhen, 518035 China; 4 Department of Plastic and Reconstructive Surgery, School of Medicine, Shanghai Ninth People’s Hospital, Shanghai Jiao Tong University, 639 Zhizaoju Road, Shanghai, 200011 China

**Keywords:** Microbiology, Diseases

## Abstract

*Acinetobacter baumannii* has become one of the most challenging conditional pathogens in health facilities. It causes various infectious diseases in humans, such as wound or urinary tract infections and pneumonia. Phage therapy has been used as an alternative strategy for antibiotic-resistant *A. baumannii* infections and has been approved by several governments. Previously, we have reported two potential phage therapy candidates, Abp1 and Abp9, both of which are narrow-host-range phages. In the present study, we screened and isolated 22 *A. baumannii* bacteriophages from hospital sewage water and determined that Abp95 has a wide host range (29%; 58/200). The biological and genomic characteristics and anti-infection potential of Abp95 were also investigated. Abp95 belongs to the *Myoviridae* family, with a G+C content of 37.85% and a genome size of 43,176 bp. Its genome encodes 77 putative genes, none of which are virulence, lysogeny, or antibiotic resistance genes. Abp95 was found to accelerate wound healing in a diabetic mouse wound infection model by clearing local infections of multidrug-resistant *A. baumannii*. In conclusion, the lytic phage Abp95, which has a wide host range, demonstrates potential as a candidate for phage therapy against multiple sequence types of carbapenem-resistant *A. baumannii*.

## Introduction

*Acinetobacter baumannii* is a gram-negative nonfermenting bacterium that is found in humans, soil, meat, and vegetables. *A. baumannii* cause a variety of infections, including approximately 10% of nosocomial infections^1–3^. This is largely due to the acquisition or upregulation of plasmids, integrons, and transposons, which makes *A. baumannii* easy to develop into multidrug resistant bacteria^4,5^. Since the 1980s, the number and types of multidrug-resistant *A. baumannii* strains have increased annually. The treatment of multidrug-resistant *A. baumannii* (MDR-AB) has become one of the most challenging clinical problems. Reliance on antibiotics alone to treat *A. baumannii* infections is virtually ineffective. A new anti-infective weapon is urgently required to replace antibiotics.

Bacteriophages are viruses that infect and lyse bacteria. Therefore, they may be used as natural antimicrobial agents. Phage therapy typically uses virulent phage to replicate and proliferate in host bacteria in order to produce a great quantity number of progeny and eventually lyse the host bacteria. Recently, phage therapy, which was first reported 100 years ago, has been used as an alternative treatment for difficult-to-treat infectious diseases caused by multidrug-resistant pathogens. Phage therapy has been used for the treatment of various infections, including wound infections^6,7^, osteomyelitis^8^, chronic prostatitis^9^ and pneumonia^10^. As reported, bacteriophages have several advantages compared to antibiotics. First, high specificity of the phages for the host that does not damage normal microbiota. Second, increased concentration at the infected site (due to self-multiplication in the presence of the host), which reduces the initial dose requirement. Third, ability to be rapidly distributed in various organs throughout the body (e.g., prostate, bone, etc.)^11^.

In our previous study, we successfully isolated the phage Abp1, a member of family *Podoviridae,* from hospital sewage. Its therapeutic effect was tested on a mouse model of local and systemic infection^12^. We further isolated phage Abp9, a member of *Myoviridae* family, active against drug-resistant ABzy9, which was obtained from the femoral venous catheter of a patient^13^. Abp9 was demonstrated to effectively destroy biofilm and reduce the mortality rate of rats infected with *ABzy9 i*solates. However, both Abp1 and Abp9 showed a narrow host range. In the present study, we screened and isolated 22 *A. baumannii* bacteriophages from hospital sewage water and identified one that showed wide host range (29%; 58/200), namely Abp95. These 58 strains belong to different genotypes, and most of them were carbapenem-resistant. The biological and genomic characteristics and anti-infection potential of Abp95 were also investigated.

## Materials and methods

### Collection and characterization of *A. baumannii* strains

A total of 200 *A. baumannii* isolates were collected and characterised in our previous study. All isolates were identified as *A. baumannii* via OXA-51 gene amplification and 16S rRNA gene sequencing^14^. Seven genes, namely, *gltA*, *gyrB*, *gdhB*, *recA*, *cpn60*, *gpi and rpoD*, were amplified using PCR and sequenced for multilocus sequence typing analysis^15^. To determine the locus number, we compared the sequencing results for each of seven genes, with the submitted sequences in the database. According to the sequences of seven locus numbers we obtained, the sequence types (STs) of isolates were determined. In case that any of the seven loci did not match to the submitted types, a new ST was proposed.

### Transmission electron microscopy (TEM) and bacteriophage isolation

A total of 22 *A. baumannii* lytic phages were isolated using a combination of 30 *A. baumannii* isolates as hosts. Briefly, the first enrichment of phages was conducted by mixing 1 L of hospital sewage water filtered through 0.22-µm filter (Millipore, USA) with 150 mL of the mix of 30 *A. baumannii* isolates (OD_60_0 approximately ≈ 1.0; 5 mL of each isolate), followed by incubatin overnight with aeration^14^. After overnight enrichment bacteria were harvested and filtered supernatant was used for isolation of phages. Each bacterial host (10 µL) was separately inoculated into soft agar (5 mL) to which 100 µL of supernatant from enrichment was added and poured out to form a double-layer plate to collect as many bacteriophages as possible from the sewage water filtrate. After incubation at 37 °C for 12 h, the appearance of the phage plaques in the soft agar of the plates carrying the host strains was analysed. For primary analysis of host range, each of the 22 phages purified from the plaques (resuspended in SM buffer and treated with chloroform) were dropped on the surface of double-layer plates inoculated with different *A. baumannii* host strains. As Abp95 showed the widest host range compared to the other isolated phages, it was selected for further analysis. Pure Abp95 phage particles were prepared via CsCl gradient ultracentrifugation^16^. CsCl-purified Abp95 phage particles, with a concentration of 10^11^ plaque-forming units (PFU)/mL, were allowed to adsorb for 15 min before being deposited on a carbon-coated copper grid. The phage particles were negatively stained with 2% (w/v) potassium phosphate and observed under a transmission electron microscope. The phage size (length and width) was measured using ImageJ. Phage genome kit (Norgen Biotek Corp., Canada) was used for isolation of phage DNA.

### One-step phage growth assay and thermal stability of Abp95

To construct the one-step growth curve, the phage Abp95 was added to the host bacteria *A. baumannii AB*_*2013-95*_ at the mid-exponential phase, followed by incubation at 25 °C for 15 min. Next, the mixture was harvested via centrifugation (13,000×*g* for 30 s) to remove the non-absorbed phages, after which the supernatant was discarded and the mix of *A. baumannii* host and phages was incubated at 25 °C with shaking. Fifty microliters of these cultures were collected every 20 min, and the double-layer plate method was used to determine the phage titre. To determine the thermal stability of bacteriophage Abp95, we incubated 50 μL of CsCl purified Abp95 in SM buffer at a temperature gradient (20–70 °C) for 10 min, and then tested its ability to form plaques using the double-layer plate method.

### Host-range determination and phage adsorption

As described previously^14^, 200 μL of 10^8^ CFU/mL host bacterial solution was mixed with 4 mL of melted 0.6% semi solid agar medium. This mixture was then poured onto the prepared double-layer agar medium. After cooling at room temperature for 5 min, 5 μL of Abp95 (10^9^ PFU/mL) was added onto the centre of the double-layer plates, each carrying a different *A. baumannii* host strain. The host bacteria analyzed included 200 *A. baumannii* clinical strains, *P. aeruginosa* strain PAO1, and *E. coli* strains DH5α and JM109. The plates were incubated at 37 °C for 12 h and then observed for formation of lytic plaques.

*AB*_*2013-95*_ was infected with phage Abp95 at multiplicity of infection of 0.001. Sample (1 ml) was collected every 1 min for a total of 10 min. Next, the supernatant was harvested by centrifugation (10,000×g for 1 min) to remove bacteria and adsorbed phage. After doubling the dilution, we determined the titres of the unabsorbed phage in each sample by the double-layer plate method.

### Genome sequencing of phage Abp95

The sequencing library from total DNA of phage Abp95 was generated using the TruSeq DNA sample preparation kit (Illumina, USA) and the Template Prep kit (Pacific Biosciences, USA). Genome sequencing utilizing next-generation sequencing (NGS) technology based on the Illumina NovaSeq sequencing platform was conducted to sequence these libraries with double ends (paired-end, PE) by Personal Biotechnology Company (Shanghai, China). SPAdes^17^ and A5-miseq^18^ were used to build scaffolds and contigs for data assembly. The sequencing data obtained from the Pacific Biosciences platform were assembled using Canu software^19^. All the results were integrated to generate a complete phage genome sequence.

### Bioinformatic analysis

Open reading frames (ORFs) were predicted using GeneMark (version 4.32; http://topaz.gatech.edu/GeneMark/), and the functions of the proteins encoded by the ORFs were predicted using BLASTp (https://blast.ncbi.nlm.nih.gov/Blast.cgi?PAGE=Proteins). Glimmer 3.02 was used for gene prediction^20^. tRNAscan-SE^21^, RNAmmer^22^ and Rfam were used for identifying ribosomal RNA , transfer RNA^23^, ribosomal RNA, and other noncoding RNAs, respectively. The whole genome was submitted to the National Center for Biotechnology Information (NCBI; https://www.ncbi.nlm.nih.gov/genbank/; accession number: MZ618622.1). A phylogenetic tree was constructed based on the comparison of whole genome sequences using the Molecular Evolutionary Genetic Analysis software (version 7.0). The virulence factor database (VFDB), the Comprehensive Antibiotic Research Database (CARD) and NCBI were used to search and analyze virulence, antibiotic resistance and lysogeny genes.

### Phage therapy in a diabetes wound infection mouse model

Streptozotocin (120 mg/kg body weight) was administered intraperitoneally to 6-weeks-old males C57 mice (n = 36) after 8 h of fasting. Blood glucose was measured on days 3 and 7; a blood glucose level greater than 16.8 mmol/L indicated the successful establishment of the diabetes mouse model. After feeding the mice for 1 week, the mouse model for local infection could be established. Each mouse was intraperitoneally injected with cyclophosphamide (150 mg/kg body weight) 24 h before establishing a local infection mouse model. The mice were anesthetised with sevoflurane, followed by removal of the hair on the back and buttocks of the rats with depilatory cream. A skin biopsy punch was used to create a diameter of 1 cm full-thickness skin wound on the hip of each mouse. Phosphate-buffered saline (PBS; 5 µL) containing 1.0 × 10^6^ *AB*_*2013-95*_ cells was used to infect the wounds, which were air-dried afterwards for 5 min. Twenty-four hours after infection, the mice were randomly divided into two groups. In the experimental group, 5 μL of Abp95 (1.0 × 10^9^ PFU) was applied to the wounds of the mice, while in the control group, 5 µL of PBS without phage was used as a negative control. At day 3 after infection, phage application was repeated. The wound sizes were measured and recorded on days 1, 3, and 7.

### Ethics statement

All animal experiments were conducted in compliance with the International Guiding Principles for Biomedical Research involving Animals (1985) the ARRIVE guidelines. All animal experiments were approved by the Laboratory Animal Welfare and Ethics Committee of Zunyi Medical University (approval number: 2019-2-032).

### Statistical analysis

One-way analysis of log-rank test (Mantel-Cox) or variance was used to analyse the data. Significance was set at *P* < 0.05.

## Results

### Abp95 is a lytic phage

**Figure 1:**
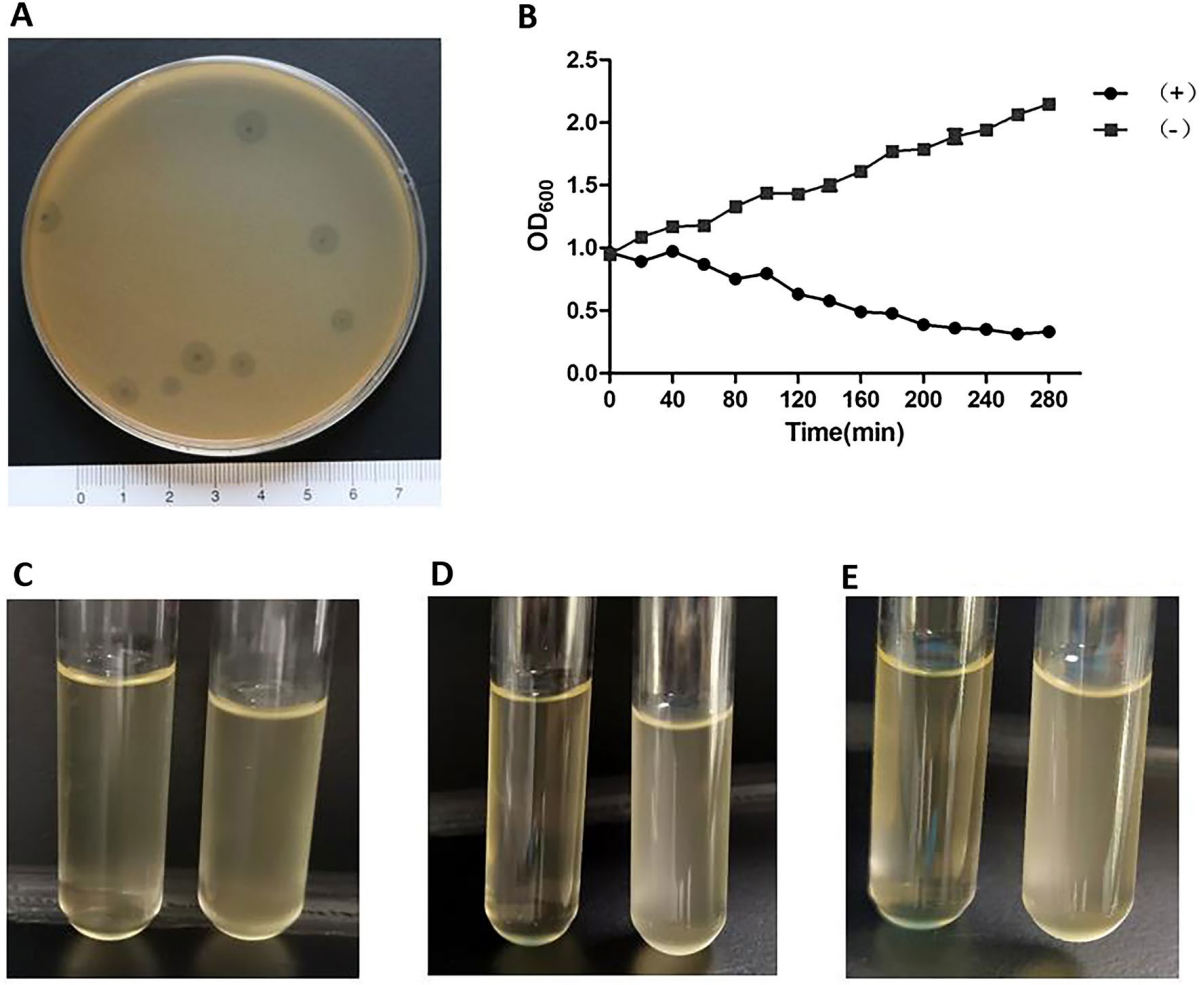
Lytic activity of Abp95 phage on A. baumannnii sensitive strain *AB*_*2013-95*_. (**A**) Abp95 plaques formed in a double-layer agar plate after infection of the *AB*_*2013-95*_ host; (**B**) Graphic of measured OD_600_ values of *AB*_*2013-95*_ bacterial culture during the time with (+) or without (−) Abp95; (**C**)–(**E**) Abp95 cause lysis in a bacterial culture after incubation. (Phage was added to the left tube and equal PBS was added to the right tube, (**D**): 4 h after adding phage, (**E**): 6 h after adding phage).

As shown in Fig. 1A, Abp95 formed plaques with big turbid halos around the plaques on the double-layer agar medium infected inoculated with *AB*_*2013-95.*_ Compared with the negative control group, the OD_600_ value of the bacterial culture (*AB*_*2013-95*_) infected with phage Abp95 dropped from 1.0 to 0.3 after 4 h (Fig. 1B). After 4 h of incubation, the bacterial culture had become completely clear as the bacteria were lysed by the Abp95 phage. After 6 h of incubation, turbidity of the bacterial culture was not observed (Fig. 1C–E).

### One-step growth curve, thermal stability and phage adsorption

**Figure 2:**
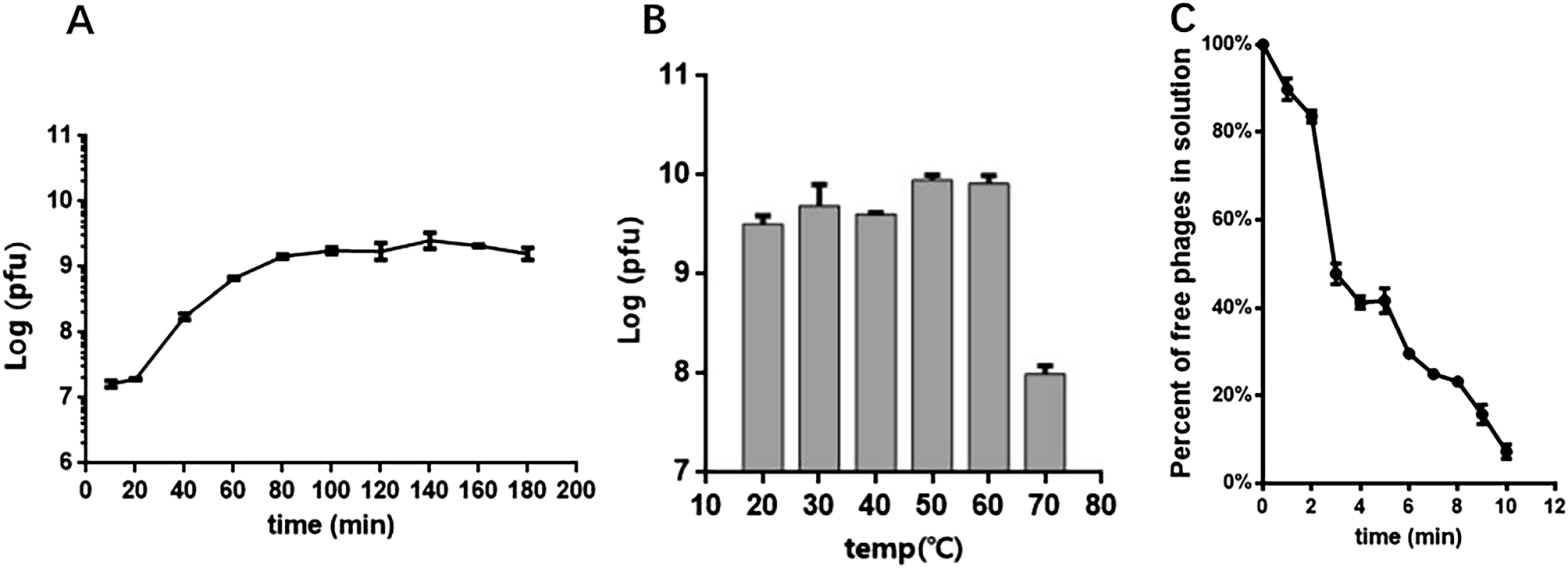
One-Step growth curve (**A**), thermal stability of Abp95 (**B**) and Phage Abp95 adsorption onto AB2013-95 (**C**).

As shown in Fig. 2A, the Abp95 phage had a latent phase of 20 min and began to burst 40 min after infection. After 120 min, the phage produced the maximum number of progenies, reaching its plateau phase. The burst size of Abp95 was 167 PFU per infected cell.

As shown in Fig. 2B, Abp95 still maintained relative activity at 60 °C, and no significant difference in phage titre was observed after treatment at 20, 30, 40, 50, and 60 °C. However, after incubation at 70 °C for 15 min, the phage titre decreased by approximately 100 folds. These results showed that Abp95 was able to maintain optimal activity at temperatures between 20 and 60 °C, making its storage and transport more convenient.

As shown in Fig. 2C, approximately 52% of the Abp95 particles were adsorbed onto *AB2013-95* within 3 min. After 10 min, approximately 93% of the Abp95 particles were adsorbed onto *AB2013-95*.

### Transmission electron microscopy

**Figure 3:**
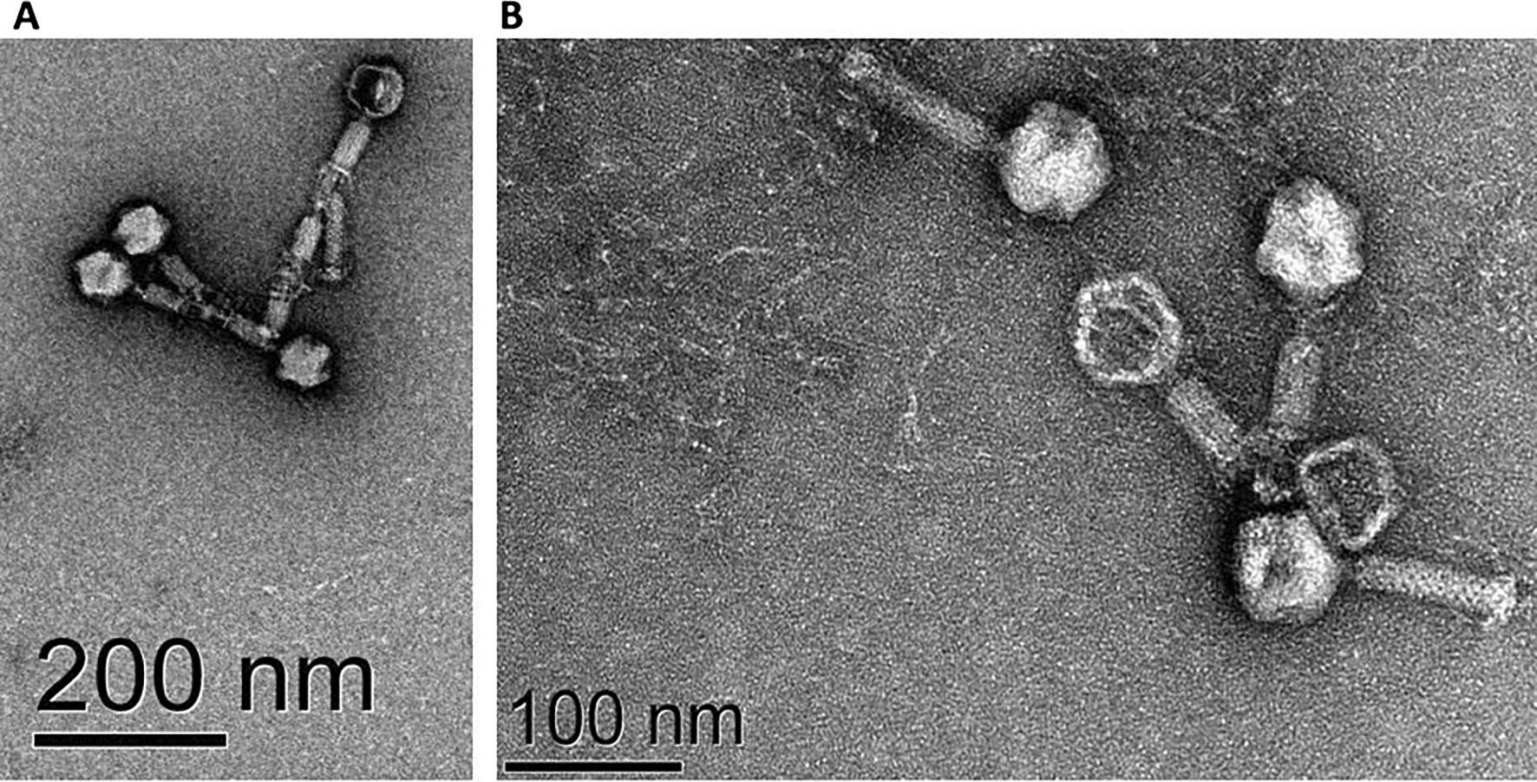
Electron microscopy of Abp95. (**A**) The morphology of Abp95 under TEM magnified by 0.5 million times (**A**) and 1 million times (**B**).

As shown in Fig. 3, Abp95 belongs to the *Myoviridae* family, and has an icosahedral-shaped head with a diameter of 60.502 ± 2 nm. Its overall length measures (142.891 ± 4.32) × (24.53 ± 2.24) nm, and the tail measures 54.752 ± 2.487 nm. Both the contraction and relaxation states of Abp95 tail were observed via TEM.

### Major structural proteins and genome structure analysis of Abp95

**Figure 4:**
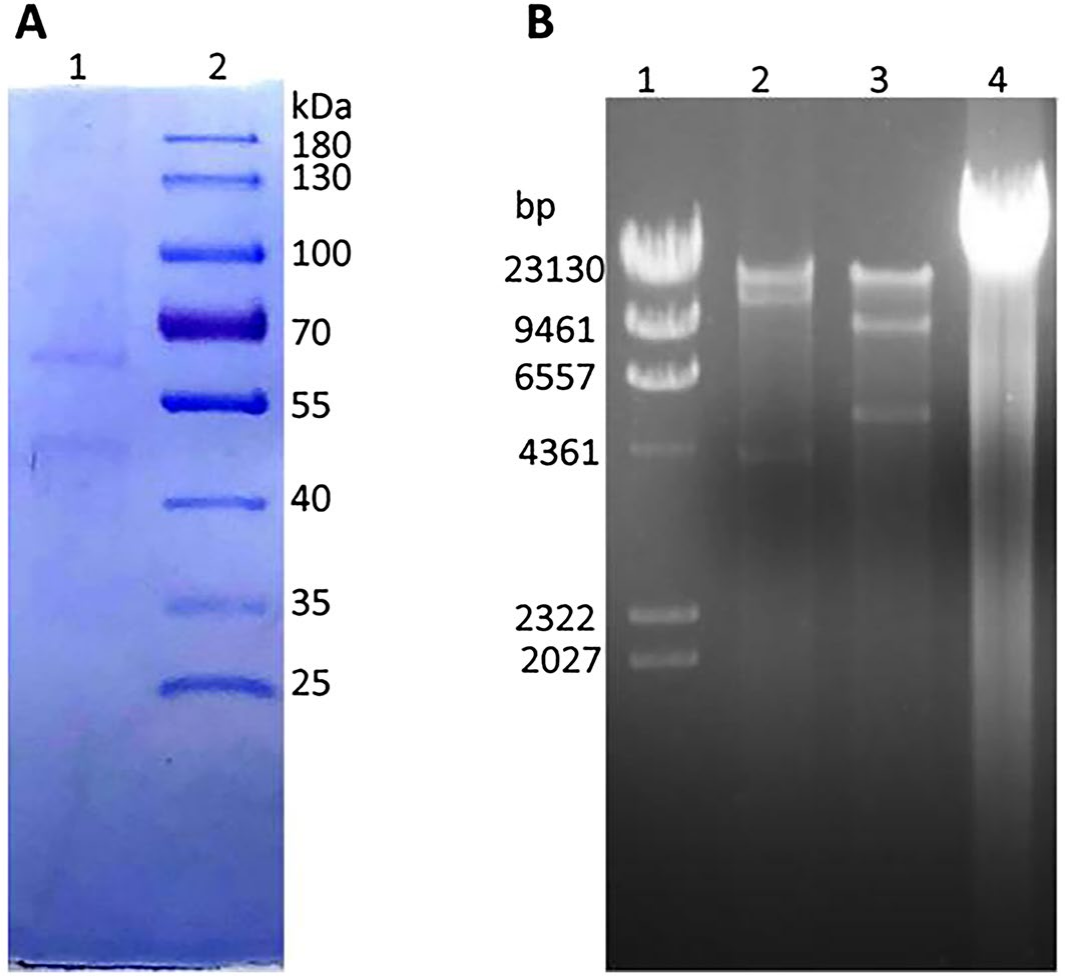
Major phage proteins and restriction endonuclease mapping of Abp95. (**A**) lane 1, SDS-PAGE of purified phage particles; lane 2, protein molecular weight marker. (**B**) Lane 1, DNA molecular weight markers Lambda DNA digested with HindIII; lane 2 and 3, Abp95 genomic DNA digested by XhoI and NheI, respectively; lane 4, Abp95 nondigested DNA. (the gel is cropped).

Protein sodium dodecyl sulphate–polyacrylamide gel electrophoresis revealed two major phage structural proteins, a large and small protein, approximately 60 kDa and 45 kDa in size, respectively (Fig. 4A). To determine whether the Abp95 genome is circular or linear, we conducted restriction analysis of the Abp95 genome using *Xho*I and *Nhe*I restriction enzymes. As shown in Supplementary Information 4, both *Xho*I and *Nhe*I have three restriction sites each in the Abp95 genome. Digestion of the genome with each of these two enzymes would produce four fragments if the genome is linear, while digestion of the genome would only produce three fragments if the genome is circular. The observed patterns (Fig. 4B) matched with the simulated electrophoretic patterns of *Xho*I and *Nhe*I digestion for a circular genome, indicating that the Abp95 genome was circular.

### General features and sequencing results of the Abp95 genome

Whole genome sequencing of Abp95 was performed; 8,539,910 reads and 29,484 genome coverage were obtained after quality control. Genome assembly produced a 43,176-bp dsDNA molecule with a G+C content of 38.07%. The ORF finder was used to search for ORFs larger than 100 bp, resulting in a total of 77 ORFs. The average length of the genes was approximately 560 bp, and the ORF density was approximately 1.78 genes per kb. Of the 77 ORFs, 10 were on the negative strand while the other 67 were on the positive strand. Nucleotide blast in NCBI revealed that 69 of the 77 ORFs had similar ORFs, and the functions of the proteins encoded by 14 ORFs were predicted using bioinformatics analysis. The genome sequence was deposited in the NCBI genome database under the GenBank accession number MZ618622.1. Comparative genome identity using BLAST search showed that phage Abp95 share highest identity (only certain regions but not the entire genome) with Acinetobacter phage WCHABP1 of 45,888 bp. In addition, by searching, we found that there was no virulence, lysogeny and antibiotic resistance genes present in genome of Abp95.

### Host range determination

**Figure 5:**
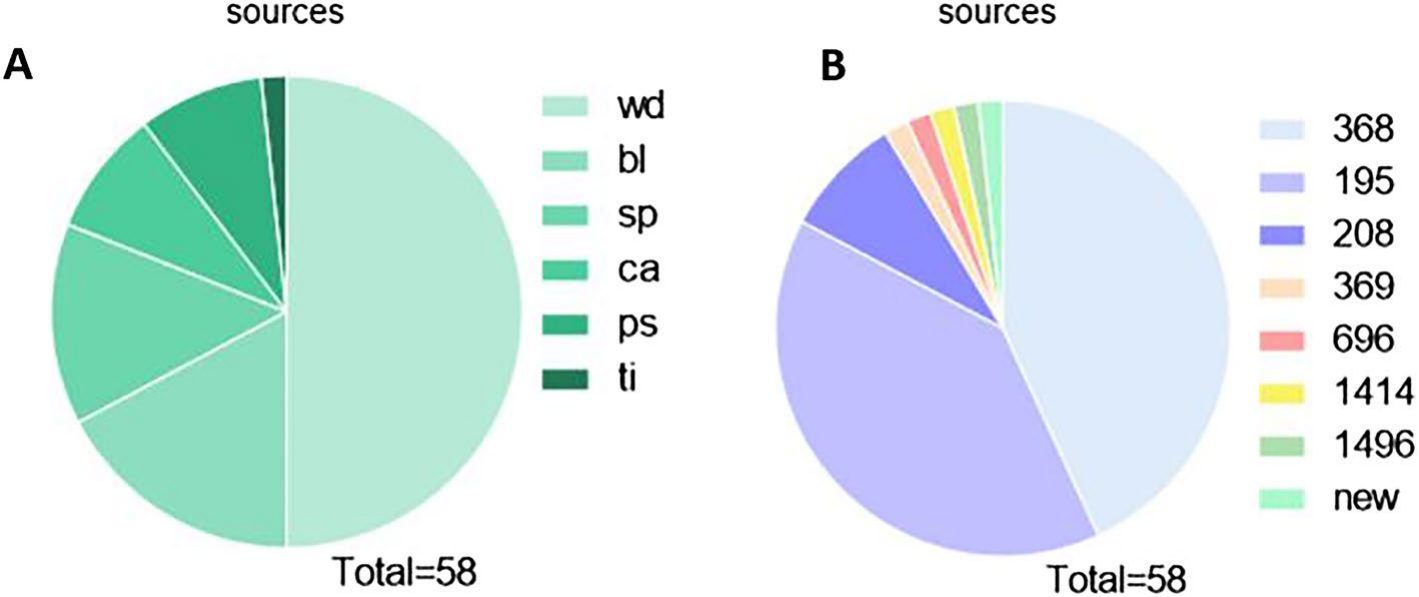
Characterization of 58 sensitive *A. baumannii* strains. (**A**) Sources of the 58 strains. wd (wound surface), sp (sputa), ca (catheter), bl (bloodstream), ps (purulent secretion) and ti (tissue); (**B**) STs identified among 58 strains.

For the host range tests, of the 200 clinical strains of *A. baumannii* tested, 58 were Abp95 sensitive, of which 55 were carbapenem resistant, while three were antibiotic sensitive. As shown in Fig. 5 and Table 1, the 58 strains were isolated from wound (29), blood (10), sputum (8), catheter (5), purulent fluid (5), and tissue (1) and belonged to eight different STs, namely, ST368 (25), ST195 (23), ST208 (5), ST369 (1), ST696 (1), ST1414 (1), ST1496 (1), and a new type (1). For the cross-species host test, Abp95 was resistant to infection with P. aeruginosa (PAO1) or E. coli (JM109 and DH5α).

**Table 1:** Host-range of Abp95.

Isolate number	Origin	ST type	Lysis effect	Antibiotic resistance
2013-003	bl	195	++++	CRAB
2013-004	ca	195	++	CRAB
2013-007	ca	195	+++	CRAB
2013-011	sp	195	++	CRAB
2013-020	ca	195	++	CRAB
2013-021	wd	208	++	CRAB
2013-026	wd	195	+++	CRAB
2013-034	wd	368	+	CRAB
2013-037	wd	195	++	CRAB
2013-038	bl	368	+++	CRAB
2013-041	ps	195	+	CRAB
2013-046	wd	368	+++	CRAB
2013-047	wd	208	+	CRAB
2013-051	wd	368	+	CRAB
2013-052	wd	368	+++	CRAB
2013-054	wd	195	++	CRAB
2013-057	wd	208	++	CRAB
2013-058	wd	1414	++	CRAB
2013-064	bl	208	++	CRAB
2013-065	wd	368	+++	CRAB
2013-069	sp	368	++++	CRAB
2013-072	sp	368	++++	CRAB
2013-082	wd	368	+++	CRAB
2013-083	wd	368	++++	CRAB
2013-088	bl	368	++++	CRAB
2013-089	wd	new	++++	
2013-094	ps	368	+	CRAB
2013-095	wd	696	++++	
2013-107	ps	195	++++	CRAB
2013-111	bl	195	+++	CRAB
2013-114	bl	368	+++	CRAB
2013-119	wd	1496	++++	
2013-125	wd	368	++++	CRAB
2013-129	sp	195	++++	CRAB
2013-131	bl	368	++	CRAB
2013-143	wd	195	+++	CRAB
2013-144	ti	368	+++	CRAB
2013-157	wd	368	++++	CRAB
2013-158	wd	368	+++	CRAB
2013-159	ps	195	++++	CRAB
2013-161	wd	368	+++	CRAB
2013-165	ps	195	++	CRAB
2013-166	wd	195	+++	CRAB
2013-167	wd	195	+++	CRAB
2013-169	ca	195	+++	CRAB
2013-171	wd	195	++	CRAB
2013-174	bl	195	+++	CRAB
2013-177	ca	195	+++	CRAB
2013-181	wd	195	+++	CRAB
2013-183	sp	368	++++	CRAB
2013-188	wd	195	+++	CRAB
2012-107	wd	208	++	CRAB
2012-108	sp	368	+	CRAB
2012-110	sp	369	++	CRAB
2012-085	bl	368	++++	CRAB
2012-090	sp	368	+++	CRAB
2012-094	wd	368	++++	CRAB
2012-096	bl	368	++++	CRAB

The classified is according to the size of abp95 plaque; +: sensitive(1–3 mm) ++: General sensitive (3–6 mm) +++: Particularly sensitive (6–9 mm) ++++: Extremely sensitive(> 9 mm).

### Phage therapy in a mouse diabetic wound infection model

**Figure 6:**
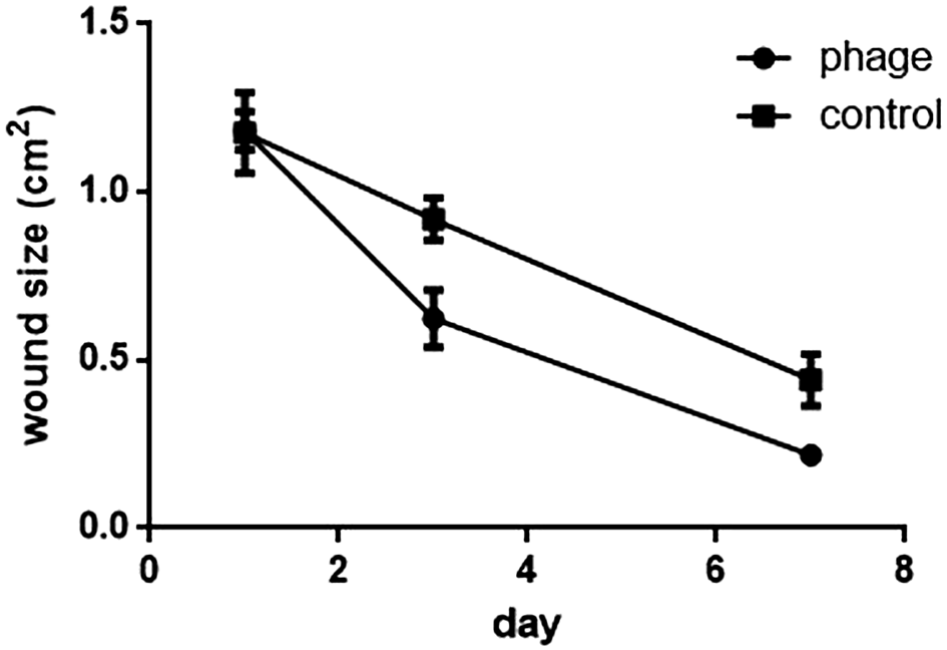
Wound size in infected mice treated with Abp95. Wound sizes were measured 1, 3, and 7 days after infection and are shown as mean ± standard deviation. There was no statistical difference between the control group and the phage treatment group.

To investigate the effects of Abp95 on infected diabetic wounds, a diabetic mouse model for local *A. baumannii* infection was established. On days 3 and 7 after infection, the areas of the phage-treated wounds were significantly smaller than those of wounds treated with PBS (Fig. 6). During a two-week treatment, no mouse died in either group. These results suggest that Abp95 local therapy not only accelerates wound healing but also has the potential to treat local infections of multidrug-resistant *A. baumannii* in clinical application.

## Discussion

Currently, polymyxin-based regimen is the most frequently used regimen for the treatment of drug-resistant *A. baumannii* (DTR-AB) infections. Two new antimicrobials, cefiderocol and eravacycline, have recently been approved and have been shown to be effective against DTR-AB. Recent clinical trials have shown that cefiderocol may serve as a potential treatment option for patients with DTR-AB; however, cefiderocol still has a higher mortality rate compared with eravacycline^24–26^. Meanwhile, eravacycline is more commonly used for abdominal infections^27^.

In contrast to the slow progress of antibiotics, bacteriophage therapy has made good progress. Studies have reported that phages have been used in treating multidrug-resistant *P. aeruginosa* and pandrug-resistant *Achromobacter xylosoxidans* infections after lung transplantation^28,29^, *Klebsiella pneumoniae* infection after artificial knee replacement and *A. baumannii* infection in patients with COVID-19^10,30^. Clinical trials of phage therapies for patients with urinary tract infections after transurethral resection of the prostate and for those with *P. aeruginosa* infection in burn wounds have been carried out^31,32^. Moreover, engineered phages have been used for the treatment of a patient with disseminated drug-resistant *Mycobacterium abscessus* infection^33^. In addition to using phage therapy alone, scientists are trying to combine phage and antibiotic treatments, and this combination has been demonstrated to inhibit resistance in bacteria. Moreover, several studies have demonstrated the synergistic effect of combined phage and the antibiotic therapy^34–37^. Few studies had focused on isolating new bacteriophages with a broad spectrum^38–40^ and developing phage cocktails^41^against DTR-AB. Meanwhile, phage encoded endolysins Ply6A3^42^, LysSS, and vB_AbaP_D2 and depolymerase enzymes have been reported to display wide antibacterial spectra^43,44^.

Approximately 25% of the people with diabetes develop diabetic foot ulcer, and more than half of them develop bacterial infections. In infections of diabetic foot ulcer, while *Staphylococcus aureus* accounts for 42% of all the cases^45^, *A. baumannii* is becoming one of the main bacteria contributing to these infections^46^. Patients with diabetes suffer from shortage of tissue perfusion and limb necrosis due to microvascular disease, which reduces local antibiotic concentrations in the skin and soft tissues^45^. At the same time, due to the overuse of antibiotics, drug-resistant bacteria are surviving and reproducing in such wounds. At present, the research and development of new antibiotics is significantly slower than the emergence of drug-resistant bacteria, and the ability of antibiotics to treat infected wounds is increasingly weaker. Phage therapy, which has been used as an alternative strategy for treatment, has intrinsic advantages, including high specificity, a low dosage requirement, no negative effect on the microbiota, and amenability to individualised treatment. Furthermore, phages can proliferate locally and increase in number, thereby being more likely to play an antimicrobial role in local wounds.

Abp95 Acinetobacter baumannii phage has a relatively wide host range. We determined that 58 out of the 200 *A. baumannii* clinical isolates were Abp95-sensitive. These sensitive hosts were from six different sources and belonged to eight different STs. Although the primary host *AB*_*2013-95*_ for isolation of Abp95 is an antibiotic-sensitive strain, 55 out of 58 of these isolates were carbapenem-resistant *A. baumannii* strains*.* To date, about 17 types of wide-host-range phages that infect *A. baumannii* have been reported , and all of them can lyse MDRAB like Abp95. In the current study, the host range of seven phages exceeded 50%, and the host range of two phages, namely øABP-01^47^ and QAB 3.4^48^, was 100% (12/12 and 100/100, respectively). φABP2^49^, vB_AbaM_PhT2^50^ and Abp95 displayed host range (16/60 and 42/150); however, φABP2 displayed a shorter latent phase (15 min) and a larger burst size (222 PFU/cell). Nien-tsung Lin et al.^51^ reported that φAB2 belonged to the Podoviridae family, with a host range of 19.6% (25/127) ); however, the latent phase was reported to be only 10 min, with a large burst size (200 PFU/cell) and high adsorption rate, reaching 100% within 10 min. Although the host range of βφ-R1215^52^ and β φ-R2315^52^ was approximately 46%, the latent phase was longer (30 and 40 min, respectively), and the burst size was also smaller (43 and 78 PFU/cell, respectively). Collectively, these results indicated that although the host range of Abp95 was not broad compared with that of other broad-spectrum bacteriophages, the latent phase was shorter, the burst size was larger, and the adsorption rate was higher, suggesting that Abp95 may potentially be used as a substitute for antimicrobial agents.

At the same time, we also found that when Abp95 forms plaque, it will produce a translucent halo around it, which is the sign of the existence of depolymerase, a similar phenomenon was observed in many Acinetobacter *baumannii* phages. Depolymerase is the key to phage adsorption, which can degrade cap polysaccharide peptides (CPS), exopolysaccharide peptides (EPS), and lipopolysaccharide peptides (LPSs)^53^, thus assisting the phage to pass through the bacterial protective barrier and achieve efficient sterilization. It is reported that depolymerase has shown good effects in the treatment of bacterial infections, for it can improve the sensitivity of antibiotics^54^ and enhance the sensitivity of the complement system to serum killing^55^. The combination of depolymerase and antibiotics can effectively destroy the biofilm^56^. Not only that, depolymerase also shows certain potential in vaccine research and development^57^. Therefore, Abp95 has high scientific research value.

With satisfactory thermal stability, Abp95 is a broad-spectrum bacteriophage and shows prospect for the treatment of carbapenem-resistant *A. baumannii.* For optimizing the clinical use of Abp95, its clinical effect and rate of clearing Abp95 biofilm needs to be further evaluated. Moreover, the antibacterial effect of Abp95 combined with antibiotics needs to be explored, which will be the direction of our future research (Supplementary Information 1, Supplementary Information 2, Supplementary Information 3, Supplementary Information 4).

## Supplementary Information

Supplementary Information 1.

Supplementary Information 2.

Supplementary Information 3.

Supplementary Information 4.

## Data Availability

The datasets generated during the current study are available in the NCBI genome database repository and article, Acinetobacter phage Abp95, complete genome—Nucleotide—NCBI (nih.gov).
