# Supplementary figures and images for: Characterisation and sequencing of the novel phage Abp95, which is effective against multi-genotypes of carbapenem-resistant Acinetobacter baumannii
Source: Sci Rep. 2023 Jan 5;13:188. doi: 10.1038/s41598-022-26696-9 (PMC9813454; doi:10.1038/s41598-022-26696-9)

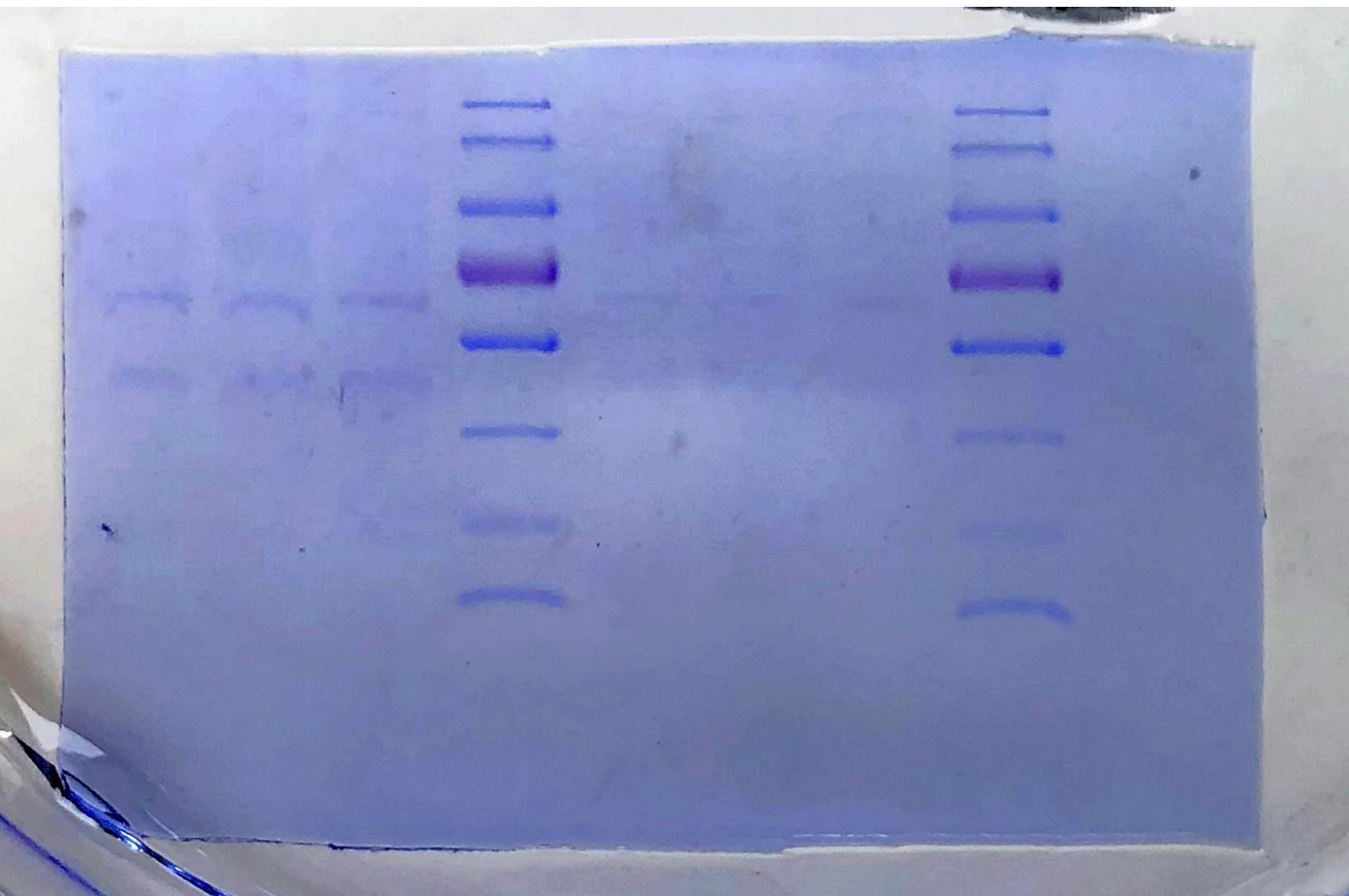

Supplement: Supplementary file 2 — Supplementary Information 2. [file 41598_2022_26696_MOESM2_ESM.pdf]

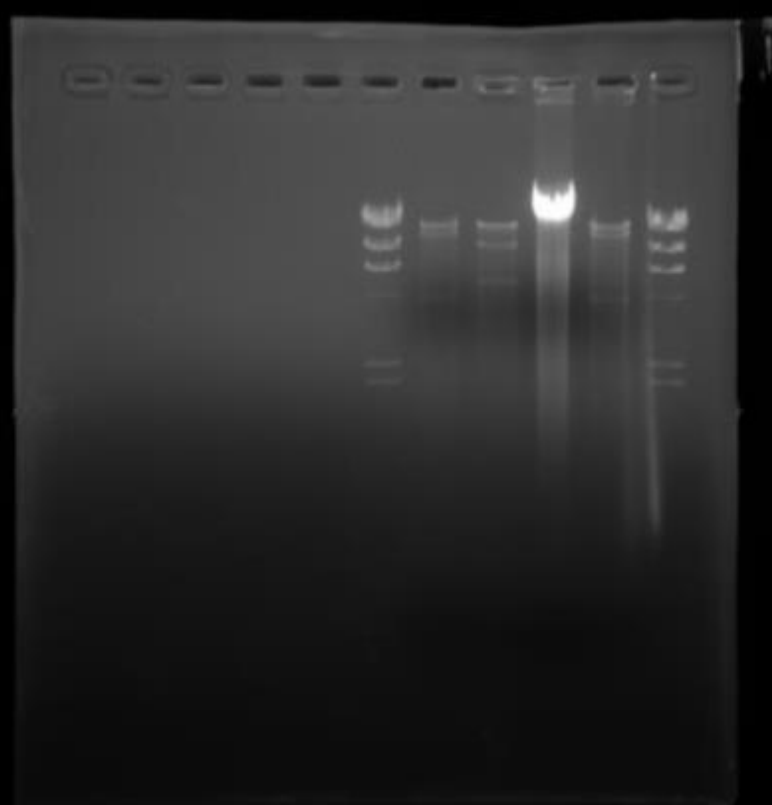

Supplement: Supplementary file 3 — Supplementary Information 3. [file 41598_2022_26696_MOESM3_ESM.pdf]
