# Supplementary material for: Characterisation and sequencing of the novel phage Abp95, which is effective against multi-genotypes of carbapenem-resistant Acinetobacter baumannii
Source: Sci Rep. 2023 Jan 5;13:188. doi: 10.1038/s41598-022-26696-9 (PMC9813454; doi:10.1038/s41598-022-26696-9)
Supplement: Supplementary file 4 — Supplementary Information 4. [file 41598_2022_26696_MOESM4_ESM.pdf]

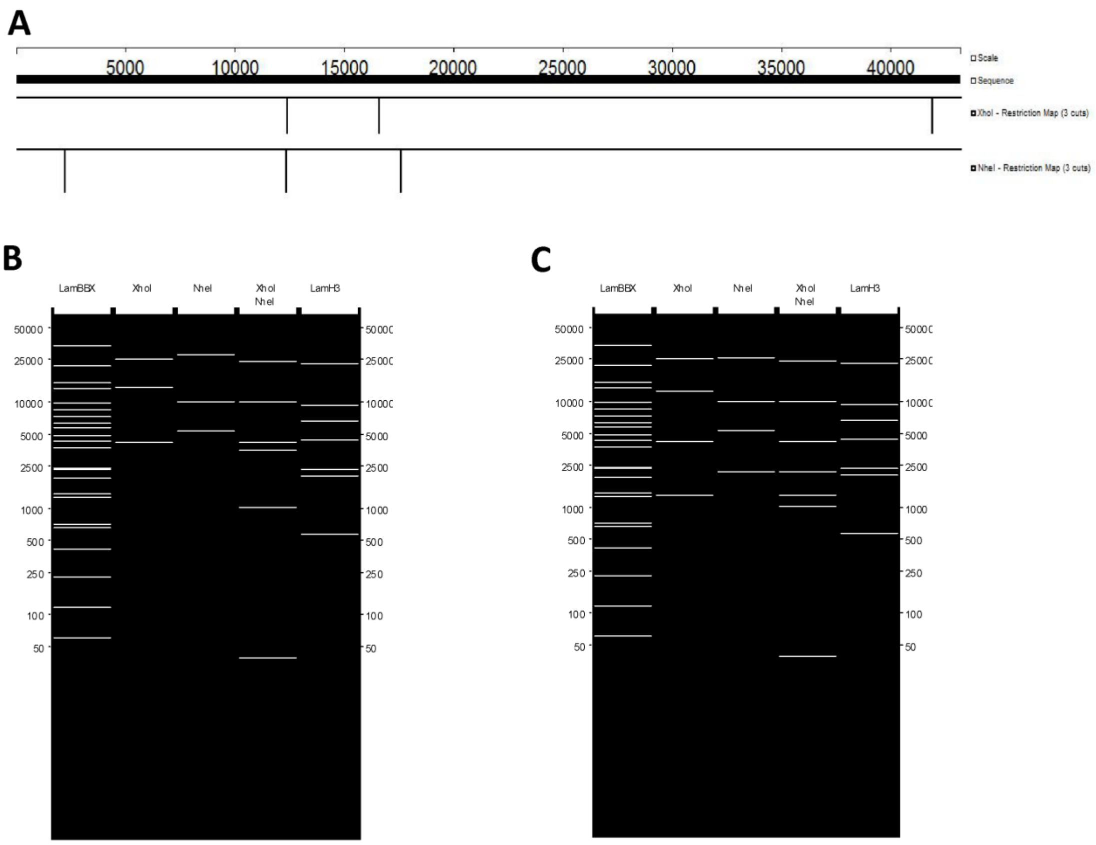

Figure S1. Restriction analysis of Abp95 genome. A) XhoI and NheI cut sites among Abp95 genome by GeneQuest. B) Simulated electrophoretic pattern of XhoI and NheI digestion for circular genome. C) Simulated electrophoretic pattern of XhoI and NheI digestion for linear genome.
